# Understanding the Motivations of Foster Caregivers at Animal Shelters

**DOI:** 10.3390/ani13172694

**Published:** 2023-08-23

**Authors:** Roxy Ackerman, Brittany Watson, James Serpell, Chelsea L. Reinhard, Lauren Powell

**Affiliations:** School of Veterinary Medicine, University of Pennsylvania, Philadelphia, PA 19104, USA; roxyack@vet.upenn.edu (R.A.); brittawa@vet.upenn.edu (B.W.); serpell@vet.upenn.edu (J.S.); creinh@vet.upenn.edu (C.L.R.)

**Keywords:** animal welfare, foster animal, foster care, human–animal bond, shelter medicine, volunteer, volunteer motivations

## Abstract

**Simple Summary:**

Foster care programs in animal shelters are important tools to help support animal welfare, reduce the risk of disease, and create more kennel space for incoming animals. However, little is known about what drives foster caregivers to volunteer to care for shelter animals in their homes. This study surveyed foster caregivers within the United States to evaluate their motivations for and expectations of the foster care experience. We found that the most common motivations for fostering were animal- and community-related. Younger caregivers were more likely to expect companionship, emotional support, and to meet other community members through fostering than older caregivers, while male caregivers were less likely to foster for emotional support compared with females. We also found that dog caregivers and those who did not own pets at home were more likely to expect companionship from their foster animal. This study provides a better understanding of the motivations and expectations of foster caregivers and hopes to aid shelters to recruit foster care volunteers more effectively in the future.

**Abstract:**

Foster care programs in animal shelters have been shown to benefit animals and shelters, but little is known about what motivates foster caregivers to volunteer. This cross-sectional study explored the motivations and expectations of 131 foster caregivers from five shelters within the United States who completed a pre-foster survey between March 2022 and March 2023. The most common motivations were animal- or community-based, such as the desire to provide an animal with love or do something positive for the community. Ordinal logistic regression models were used to investigate associations between caregivers’ motivations and their prior foster experience, pet ownership history, age, gender, and foster animal species. The 18- to 29-year-old caregivers were most likely to expect companionship (OR 5.18, 95% CI 1.79–15.04), emotional support (OR 4.25, 95% CI 1.40–12.89), and to meet other community members through fostering (OR 5.04, 95% CI 1.85–13.74). Male caregivers were less likely to foster for emotional support than females (OR 0.12, 95% CI 0.03–0.48), while dog caregivers (OR 2.23, 95% CI 1.04–4.76) and non-pet-owners (OR 2.66, 95% CI 1.17–6.05) had greater odds of expecting companionship. This study highlights the importance of animal- and community-related benefits for foster caregivers and provides useful direction for shelters wanting to increase recruitment to expand their foster care programs.

## 1. Introduction

Each year, approximately 6.3 million animals enter the shelter system in the United States. Of those, an estimated 65% are adopted into homes, 13% are returned to their owners, and 14% are euthanized, based on data from 2019 [1]. While the number of adoptions is steadily increasing, shelters still struggle to care for the millions of animals that enter their facilities annually [1]. As shelter facilities often have limited resources and space, many have developed foster care programs that function by placing animals into the homes of volunteers who provide supplemental, temporary housing [2]. Foster care programs may reduce the risk of disease spread as animals are no longer housed in the shelter environment, meaning they are exposed to less animals. Foster care can also increase opportunities for adoption through foster caregivers’ networks and open up housing in the facility for incoming animals, therefore serving as an important tool for overcrowded shelters [3]. However, to ensure they are promoting animal welfare, protecting public health, and minimizing the length of stay, shelters need to invest sufficient time to train, manage, and support foster caregivers [4]. Volunteer retention is therefore essential.

While these benefits exist at the shelter level, research has demonstrated that foster care may also be a mutually beneficial experience for both human foster caregivers and fostered animals. Companion animals have been shown to improve cardiovascular and mental health in humans by means of increased physical activity and by providing a strong emotional human–animal connection [5,6,7]. At the same time, foster care has been associated with significant decreases in dogs’ cortisol:creatinine ratios, a marker that is widely used to evaluate stress in animals [8]. Foster care can also lead to improved behavioral outcomes in dogs compared with those housed in shelters, where animals kept for prolonged periods are more likely to develop new behavioral problems and exhibit socio-cognitive declines [9]. Cats can also benefit from foster care programs. In one study, removing cats from the stress and confinement of a shelter and placing them into a foster home with additional enrichment was shown to promote better well-being for fostered felines [10]. Foster care programs therefore have the potential to promote a better quality of life for both the foster animal and the human caregiver.

The animal welfare industry has long relied on volunteers to support their operations, including animal care, adoption events, fundraising, community outreach, and behavioral modification [11]. While it is unclear exactly how many people volunteer for animal welfare organizations, volunteering is generally popular across various age groups and communities with an estimated 30% of Americans formally volunteering for an organization between 2018 and 2019 [12]. In the absence of data about shelter foster caregivers, research about the motivations of volunteers in human-directed work can inform our understanding of foster care volunteerism. An Australian study conducted in 2016 found that volunteers were motivated by both “self-oriented” motivations (i.e., personal benefits) and “other-oriented” motivations (i.e., improved outcomes for others). Volunteers with other-oriented motivations were more likely to have increased well-being and satisfaction with the experience and were more likely to volunteer again [13]. Similarly, in De Maeyer et. al, human foster caregivers’ motivations were classified as either child-centered, self-oriented, or society-oriented, and researchers found that the vast majority of child caregivers were classified as having child-centered motivations [14].

More recently, three studies have investigated the experiences of foster caregivers at animal shelters [15,16,17]. One found that foster caregivers’ most common motivation concerned helping dogs and saving dogs’ lives, followed by their love or passion for animals [15]. Reese et al. evaluated the benefits of foster care programs through a national survey of foster caregivers across the United States, placing an emphasis on the necessity for animal shelters and the importance of investing in resources to make them successful [16]. Last, a 2022 study based out of Los Angeles County focused on ways to increase community engagement in shelter foster programs and found that the greatest barrier to reaching their local community pertained to non-English-speaking individuals. The researchers suggested that developing marketing materials and communications in multiple languages could help to boost engagement, emphasizing the importance of addressing local communities’ needs to increase participation [17]. While each of these recent studies addresses different components of foster care, there is still a lack of understanding regarding the foster care experience as a whole, what drives someone to participate, and what drives individuals to continue to participate.

It is important for shelters to understand the different animal- and community-related impacts of foster care programs and to be informed of the factors that contribute to a positive or negative foster care experience to increase recruitment and retention of foster caregivers and support the development of foster programs. This study aimed to evaluate the motivations of foster caregivers through the distribution of a questionnaire at five animal shelters within the United States. We hypothesized that foster caregivers will be mostly driven by animal-centered motivations (i.e., to provide a good home for an animal, to provide an animal with love, and to reduce an animal’s risk of euthanasia), similar to the findings found in human foster care studies and volunteer motivations in general [13,14,15]. Another aim of this study was to determine whether motivations were impacted by demographic variables (i.e., age, gender, and personality type) and the characteristics of foster care (i.e., type of species fostered and previous foster experience). Based on previous research about volunteers’ motivations [18], we hypothesized that younger foster caregivers would be motivated by both personal benefits and altruistic values, while older foster caregivers would be more focused on altruistic motives, such as the benefits for shelter animals and the community. We also hypothesized that individuals’ motivations for foster caregiving may differ between dog and cat caregivers as previous research has found dog adopters have greater expectations for the human–animal relationship, animal behavior, and the effort required in pet ownership compared with cat adopters [19]. Finally, we predicted that previous foster care experience may impact caregiver expectations as adopters with increased animal care knowledge have reported greater expectations for the amount of effort required in pet ownership [19]. 

## 2. Materials and Methods

### 2.1. Study Population

Foster caregivers were recruited from five different shelters within the United States: Dakin Humane Society (DHS), Providence Animal Center, Humane Animal Partners (HAP) (representing the partnership between Delaware Humane Association (DHA) and Delaware Society for the Prevention of Cruelty to Animals (DSPCA), which occurred partway through our study), Massachusetts Society for the Prevention of Cruelty to Animals (MSPCA), and San Diego Humane Society (SDHS). These shelters were included opportunistically in an effort to gather the largest sample size possible for the study. A breakdown of each shelter’s location, number of active sites, number of companion animals admitted per year, and number of valid responses is provided in Table 1. The eligible participants included those who agreed to provide temporary care to a shelter animal in their home, were over 18 years old, and were not planning to use the foster period as a trial adoption for the fostered animal. We employed a seven-day wash-out period, meaning foster caregivers were only eligible to participate if they had not fostered an animal within the last seven days. This period was chosen to reduce the risk of caregivers’ having biased expectations from recent foster experiences, while also ensuring that we were able to recruit an adequate sample including repeat foster caregivers. All participants provided written informed consent. This study was reviewed and approved by the University of Pennsylvania Institutional Review Board (protocol number 850635). 

### 2.2. Study Design

We surveyed foster caregivers between March 2022 and March 2023. The questionnaire was conducted on Qualtrics at the shelter using a provided iPad or via email up to seven days before the foster animal was taken home, depending on the availability of iPads and the shelter’s internal processes. For the latter, shelters sent the survey link to the foster caregiver in an email after signing up for an appointment to pick up their foster animal.

Shelters either provided direct access to their shelter records or biweekly reports of their foster caregivers and fostered animals so that we could record the foster animal’s species and the date that the animal entered foster. In the case that researchers had direct access to shelter animal records, either Shelter Luv or PetPoint were used depending on which system the shelter utilized. 

### 2.3. Survey Content

#### 2.3.1. Demographics

Individual demographics of the study population were collected in the survey, with questions pertaining to gender, age, and race or ethnicity. The survey also included five questions regarding the foster caregiver’s history of pet ownership and animal fostering as we anticipated that differences in these responses may impact the foster experience.

#### 2.3.2. Motivations

The survey had 13 questions regarding motivations for providing foster care, which were evaluated on a four-point Likert scale. For each prompt, participants noted how applicable the statement was to them by selecting either, “not at all true,” “somewhat true,” “moderately true,” or “very true.” There was also an open-ended question asking if the participant was motivated by any other reasons that were not previously mentioned in the survey. Many of these questions about foster motivations were derived from De Maeyer’s 2014 survey discussed above. However, as De Maeyer’s survey was designed for human foster caregivers, some additional prompts were included to capture other potential benefits of animal caregiving and the nuances associated with animal care, such as the concept of fostering to improve physical activity levels or to reduce an animal’s risk of euthanasia [14]. 

#### 2.3.3. Ten Item Personality Inventory (TIPI)

The Ten Item Personality Inventory (TIPI) is a 10-item questionnaire that evaluates human personality based on a five-dimensional personality framework: extraverted/enthusiastic, agreeable/kind, dependable/organized, emotionally stable/calm, and open to experience/imaginative [20]. Foster caregivers were prompted to indicate how applicable each descriptor was to them on a seven-point scale from “disagree strongly” (1), to “neither agree nor disagree” (4), to “agree strongly” (7). Each dimension was presented with two descriptive prompts, and each pair of adjectives was appropriately reversed and then averaged to generate scores for each personality trait. The TIPI is both valid and reliable and has been used within similar populations of adult dog owners [20,21]. 

### 2.4. Statistical Analysis

All statistical analyses were conducted in IBM SPSS Statistics, version 28.0. First, we used the Mann–Whitney U test and independent samples median tests to select variables that appeared to be associated with foster caregivers’ motivations (*p* < 0.20). We then ran ordinal logistic regression models with backward stepwise elimination to predict foster caregivers’ motivations based on their demographic characteristics, personality, and previous foster experience for all motivations with sufficient variability in the sample. The assumption of proportional odds was assessed using a full likelihood ratio test to compare the fit of each proportional odds model with a model with varying location parameters. Multicollinearity was assessed based on Variance Inflation Factor (VIF) values with any values greater than 5 removed. Pet ownership was included as a binary variable (current owner/non-owner). Shelter was excluded from the models due to the low sample size at some shelters and moderate levels of multicollinearity between shelters. Due to the small sample size, we also excluded foster caregivers who did not provide their age (*n* = 2) or gender (*n* = 1), who selected “other” for gender (*n* = 1), and who fostered species other than cats or dogs (*n* = 5) from the regression models. *p* values < 0.05 were considered statistically significant.

## 3. Results

### 3.1. Demographics

A total of 199 individuals completed the survey, although 68 entries were excluded because the entry was incomplete (*n* = 16), the survey was completed after the foster animal was taken home (*n* = 42), the individual had fostered an animal within the last 7 days (*n* = 3), a foster animal was taken home more than 7 days after the survey was completed (*n* = 2), a foster animal was not taken home (*n* = 3), or the individual had completed the survey more than once (*n* = 2).

Most human participants were 18–29 years old (42.0%), identified as female (89.3%) and white (82.4%), had previous pet ownership experience (91.6%), had previous experience fostering an animal (53.4%), and had a pet at the time of foster (58.8%). Among the current pet owners, most respondents owned dogs (*n* = 48, 36.6% of whole sample), although 29.0% owned cats (*n* = 38), and 16.0% owned other species (*n* = 21). Of the animals fostered, 49.6% were dogs, 46.6% were cats, and 3.8% were other species (i.e., guinea pigs and rabbits) (Table 2).

Regarding the TIPI component of the survey, conscientiousness had the highest reported median amongst foster caregivers surveyed with a median of 6.50, while extraversion had the lowest reported median (median = 5.00; Table 2). In comparison with population norms, our sample scored highly on each of the different components of the TIPI (extraversion, agreeableness, conscientiousness, emotional stability, and openness to experiences) [22]. 

### 3.2. Motivations

**Figure 1:**
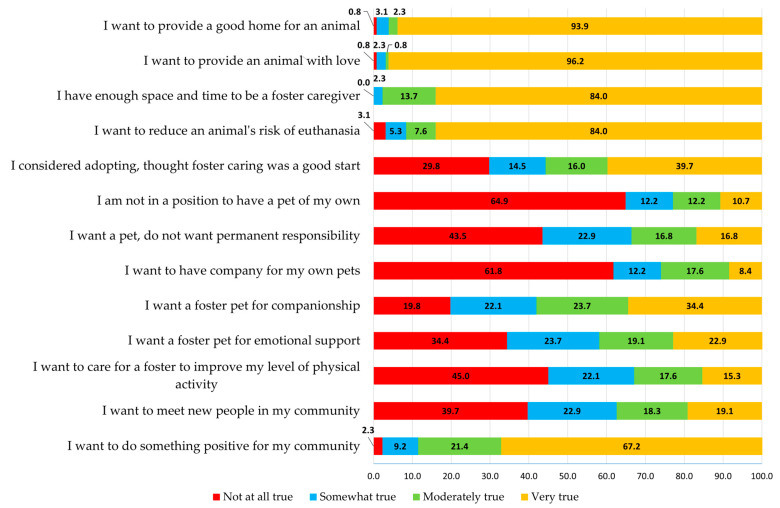
Foster caregivers’ reported primary reason for fostering on a four-point scale (not at all true, somewhat true, moderately true, and very true), represented by the percent (%) of participants that selected each motivation prompt (*n* = 131).

Motivations that centered around providing a good home for an animal, providing an animal with love, having enough time and space to provide care, reducing an animal’s risk of euthanasia, and doing something positive for the community were each highly skewed with most participants strongly agreeing with the statements (93.9%, 96.2%, 84.0%, 84.0%, and 67.2%, respectively, noted “very true”). On the other hand, motivations such as fostering a pet to avoid permanent responsibility, to improve their level of physical activity, and to meet new people in the community were each skewed in the opposite direction with many individuals indicating the selected motivation was not at all applicable (43.5%, 45.0%, and 39.7%, respectively, noted “not at all true”) (Figure 1). Generally speaking, the sample mostly identified with animal-centered motivations, as compared with self- or community-oriented motivations. Responses to the open-text question are provided in the Supplementary Materials.

The associations between foster caregivers’ motivations for providing foster care and their demographic or foster characteristics, such as age, gender, and personality, were also evaluated (Table 3). The outcome variable was collapsed into three categories, “not at all true,” “somewhat to moderately true,” and “very true,” to ensure the models met the assumption of proportional odds for ordinal logistic regression models.

Age was consistently associated with each of the motivations; most notably, those in the 18- to 29-year-old age group were about 8 times more likely to have considered adopting but thought that fostering was a good place to start, 5 times more likely to be fostering for companionship, and 5 times more likely to want to meet people in the community through fostering compared with the 50+-year-old reference group. Those who fostered dogs were more than 7 times more likely to be fostering to improve their physical activity and 2 times more likely to be seeking companionship from the foster experience compared with cat foster caregivers. Additionally, people who did not own a pet at the time of fostering were 2 to 4 times more likely to be seeking companionship, emotional support, and avoiding the responsibility associated with pet ownership compared with current pet owners (OR 2.92, OR 2.66, and OR 4.08, respectively; Table 3). 

We also found significant relationships between caregivers’ personality traits and their motivations to provide foster care. For example, people who considered themselves more open were significantly more likely to be fostering to seek emotional support from their foster animal, while those who had greater levels of emotional stability were significantly less likely to seek emotional support from foster care. Also, individuals who scored higher on the extraversion subscale were more likely to have considered adopting but thought fostering was a good place to start (Table 3).

## 4. Discussion

While the COVID-19 pandemic led to a surge of foster care programs in shelters across the United States [7], a lack of research currently exists about what motivates foster caregivers to participate. This study sheds light on the general characteristics of foster caregivers and their motivations and provides animal shelters with the information needed to effectively recruit and retain foster care volunteers. Most foster caregivers were strongly motivated by animal-related concerns, such as providing a good home for an animal or housing an animal to reduce its risk of euthanasia. The importance of animal-centered benefits for volunteers has also been documented in previous research that found animal welfare volunteers were driven mostly by animal-based motivations [11]. For shelters seeking to increase their foster volunteer base, it may be beneficial to highlight the animal-related benefits of fostering during their outreach efforts, such as the decrease in stress levels and improved behavioral outcomes in shelter animals [7,8]. Our findings also mirror De Maeyer’s 2014 conclusions that human foster care volunteering was driven primarily by child-centered motivations. Respondents in both studies showed the highest level of agreement with the statement “I want to provide a good home for the animal/child” [14]. A similar study of American child foster caregivers also found 89% of volunteers were motivated by the desire to provide a good home for a child, and 90% wanted to provide a child with love [23].

A vast majority of the sample were also motivated to foster as they had the time and space to do so. Previous research has again found similar results in human foster care, where 94% of respondents indicated they were motivated to provide care as they had enough space and time [14]. In this case, the researchers concluded that individuals with more available time may have wanted to fill that time by doing something important. However, the idea that foster caregivers were motivated by their access to resources may also speak to noblesse oblige, the concept that privileged people have a moral responsibility to help those who are less privileged. While noblesse oblige can help to increase philanthropy, it also has the potential to perpetuate power imbalances in volunteering through a sense of volunteer entitlement and recipient debt [24]. To our knowledge, there is no existing scientific evidence about the incidence or impacts of noblesse oblige in animal welfare volunteering. 

Respondents showed considerably more variability in their caregiver-based motivations. Mirroring previous research about volunteerism [25], foster caregiving was most common among younger individuals aged 18–29 years and 30–39 years. Age was also consistently related to caregiver motivations. The 18- to 29-year-old age group had the greatest likelihood of seeking companionship, emotional support, and connections within their communities through the foster experience. They were also more likely to provide foster care because they considered adopting but thought foster care was a good way to start. Those in the 30- to 39-year-old age group were similarly motivated by companionship, emotional support, physical activity, and connections within their community, as well as the desire to care for a pet without permanent responsibility. These findings support our initial hypothesis and mirror some previous literature that found younger foster caregivers were motivated by both altruistic values and personal benefits, while older foster caregivers were more focused on altruistic values, namely, the benefits to shelter animals and the community [18]. Shelters should be aware of the generational differences in foster caregivers’ motivations to maximize their recruitment and retention. For example, shelters may use tailored advertisements to highlight the potential to meet individuals in the community through foster care when targeting younger age groups. Given the similarities between pet ownership and foster caregiving, shelters could also highlight evidence that shows pet owners have a heightened ability to meet members of their community through pet-related activities, such as taking their dog on walks, going to dog parks, or visiting nearby animal facilities, which can facilitate social interaction and friendship formation within their neighborhoods [26]. Additionally, as younger age groups demonstrated a strong interest in fostering, shelters may also utilize social media platforms to promote their foster care programs among these target groups [27]. 

Non-pet-owners were significantly more likely to foster for companionship or emotional support or because they did not want the responsibility associated with full-time pet ownership compared with current pet owners. Participants with pets may already receive the companionship and emotional support associated with pet ownership from their current pet, in comparison with non-pet-owners who instead are actively seeking these qualities from their new foster animal. 

There were also associations between the types of fostered species and motivations to foster. For example, individuals who fostered dogs were more likely to be fostering for companionship compared with cat foster caregivers. Studies have found that owners typically perceive dogs to be more loving, playful, sociable, and protective than cats [28]. A majority of prospective dog caregivers also expect to receive companionship from the dog [29], so it follows that those fostering dogs were more likely to seek companionship compared with cat foster caregivers. Additionally, dog foster caregivers were more likely to be motivated to foster to improve their levels of exercise. Previous literature has also found that dogs have the potential to increase their owners’ physical activity [6]. Shelters may utilize this information in foster caregiver recruitment, highlighting the known increased activity levels in pet owners to prospective volunteers.

Our sample pool scored higher on each of the five personality traits compared with population norms [22]. This finding is consistent with previous research that compared volunteering and non-volunteering populations. These studies found that people who volunteer, on average, score higher on all five scales of the TIPI—extraversion, agreeableness, conscientiousness, emotional stability, and openness to experiences—in comparison with non-volunteering individuals [30,31]. We also found differences in foster caregivers’ expectations based on their personality traits. Participants with higher levels of openness were more likely to provide foster care as they expected the foster animal would provide emotional support, whereas participants with higher levels of emotional stability were less likely to seek a foster pet for emotional support. Providing foster care can be emotionally challenging for volunteers as shelter animals often have unknown histories and unique medical or behavioral challenges. Frequent fostering, fostering special needs animals, and fostering puppies can all lead to burnout among caregivers [16]. Human foster caregivers have described feelings of grief, loneliness, and having difficulty letting go when their foster child departs the home [32]. Shelters must consider the foster caregivers’ motivations, expectations, and experience when matching foster animals with caregivers and ensure they are providing adequate support to all caregivers. In some cases, it may be advisable to place more challenging foster animals with individuals who do not purport to rely on the animal for emotional support, particularly if there is a significant risk of euthanasia.

Males were 88% less likely to be motivated to foster for emotional support. Although men and women are similar in terms of the type of emotional support they want to receive [33], research shows that men are less likely to say that they need emotional support compared with women, which may explain the difference between male and female responses in our study [34]. It is also possible that men were less likely to seek emotional support from companion animals compared with women and perhaps rely on other sources of support. A vast majority of respondents in our study were female, meaning the motivations of male foster caregivers are less reliable as they may be subject to type 2 error because of the small sample size.

There were no significant differences between first-time and repeat foster caregivers, opposing our preliminary hypothesis that previous foster experience may lead to more reasonable expectations about the benefits and challenges associated with foster care. The lack of significant differences may be due to the large proportion of the sample with previous pet ownership experience. Increased animal care knowledge is positively related to owners’ expectations about the amount of effort required in pet ownership [19], which may negate any differences related to foster experience specifically.

### Limitations

This research has some associated limitations. First, we could not calculate the response rate of foster caregivers at each shelter. Therefore, it is possible that our sample is not reflective of the entire pool of foster caregivers, as caregivers who felt more passionately about fostering may have been more likely to participate in our survey compared with those who felt indifferent to the process. Second, while the participating shelters were located across four different states, each was in the northeastern region of America except for San Diego Humane Society. Further, the participating shelters were all located in more urban environments. Because of this, our study lacks input from foster caregivers from the south and midwestern United States, as well as more rural populations, and therefore may not be representative of the more generalizable population. Third, certain variables that could impact caregivers’ expectations, such as education level, family structure, and income, were not included in the survey due to the length of the survey. Last, the sample included an overrepresentation of Caucasian respondents and females, which is not uncommon in human–animal interaction research. However, the lack of diversity in this study could also reflect homogeneity within the foster volunteer population. Previous studies have found the majority of animal welfare volunteers were female, White, heterosexual, and in full-time employment [11]. The Association of Shelter Veterinarians has recognized the need to increase diversity among shelter staff and volunteers to ensure shelter organizations are representing the communities that they serve [4,35]. Our study also illustrates the need for further research to understand the lack of diversity in animal welfare and to identify strategies to overcome barriers [36]. 

## 5. Conclusions

This study evaluated the motivations of foster caregivers in five animal shelters in the United States, based on demographic variables and the characteristics of foster care. Foster caregivers were mostly driven by animal-centered motivations, although variables, such as age, gender, personality traits, and foster species, impacted caregivers’ expectations for personal benefits from fostering. This information may help animal shelters to identify the needs and expectations of foster caregivers to increase recruitment and retention, and in the creation of new foster care programs. The findings may also help to inform shelter staff to better match foster animals with appropriate foster caregivers. However, longitudinal data are needed to understand how foster caregivers’ motivations impact their experiences while providing foster care and their willingness to provide care to shelter animals in the future. 

## Figures and Tables

**Table 1:** Description of the five shelters surveyed during the study (*n* = 131).

Shelter Name	Location	Number of Sites	Number of Animals Admitted in 2022	Number of Valid Responses
Humane Animal Partners *	Newark, DE, USA Wilmington, DE, USA	2	3377	73
Providence Animal Center	Media, PA, USA	1	3231	21
Dakin Humane Society	Springfield, MA, USA	1	2655 **	14
Massachusetts SPCA	Methuen, MA, USA Boston, MA, USA Cape Cod, MA, USA	3	5906	12
San Diego Humane Society	San Diego, CA, USA	4	29,088 ***	11

* Humane Animal Partners (HAP) represents the combination of Delaware SPCA and Delaware Humane Association, which merged partway during the data collection period. ** Excludes owner-requested euthanasia and animals who were dead on arrival. *** Excludes intakes of wildlife.

**Table 2:** Summary data table of eligible foster caregiver demographics, separated by gender, age group, race, previous experience providing foster care, whether the caregiver had a pet in their home at the time of foster, and what type of animal was fostered (*n* = 131), as well as the TIPI survey distribution, reported by median and interquartile range (25–75%).

Human Demographics		Frequency	Percentage %
Gender	Male	12	9.2
	Female	117	89.3
	Other	1	0.8
	Prefer not to answer	1	0.8
Age	18–29	55	42.0
	30–39	28	21.4
	39–49	19	14.5
	50+	27	20.6
	Prefer not to answer	2	1.5
Race	Asian	9	6.9
	Black or African American	2	1.5
	Hispanic or Latino	2	1.5
	Native Hawaiian/Pacific Islander	1	0.8
	White	108	82.4
	Mixed race	5	3.8
	Prefer not to answer	4	3.1
Foster experience	Have fostered before	70	53.4
	Have not fostered before	61	46.6
Pet ownership	Previous pet owner	120	91.6
	Current pet owner	77	58.8
	Non-pet-owner currently	54	41.2
Types of animals fostered	Dog	65	49.6
	Cat	61	46.6
	“Other”	5	3.8
		**Median**	**Interquartile range**
TIPI distribution	Extraversion	5.00	3.50–5.50
	Agreeableness	6.00	5.00–6.50
	Conscientiousness	6.50	6.00–7.00
	Emotional stability	5.50	4.50–6.50
	Openness to experiences	6.00	5.00–6.50

**Table 3:** Ordinal logistic regression models with backward stepwise elimination showing associations between caregivers’ motivations for providing foster care and their sociodemographic characteristics, personality, and foster experience (*n* = 124).

		Motivations					
		Considered Adopting	Do Not Want Responsibility	Companionship	Emotional Support	Improve Physical Activity	Meet People in Community
Variables		OR (95% CI)	OR (95% CI)	OR (95% CI)	OR (95% CI)	OR (95% CI)	OR (95% CI)
Foster Species	Dog	1.86 (0.88–3.95)	1.81 (0.88–3.70)	**2.23** **(1.04–4.76)**	-	**7.62** **(3.35–17.43)**	-
	Cat	Ref	Ref	Ref	-	Ref	-
Foster experience	Repeat foster	0.56 (0.24–1.28)	-	-	-	-	-
	First-time foster	Ref	-	-	-	-	-
Current pets	Non-owner	-	**2.92** **(1.33–6.42)**	**2.66** **(1.17–6.05)**	**4.08** **(1.72–9.71)**	2.16 (0.96–4.89)	-
	Owner	-	Ref	Ref	Ref	Ref	-
Age	18–29 years	**7.89** **(2.62–23.79)**	2.18 (0.79–6.03)	**5.18** **(1.79–15.04)**	**4.25** **(1.40–12.89)**	2.60 (0.85–7.97)	**5.04** **(1.85–13.74)**
	30–39 years	1.97 (0.68–5.73)	**3.89** **(1.31–11.56)**	**5.00** **(1.62–15.43)**	**4.77** **(1.47–15.46)**	**4.26** **(1.29–14.03)**	**3.17** **(1.03–9.73)**
	40–49 years	1.04 (0.31–3.51)	0.92 (0.25–3.40)	2.85 (0.82–9.85)	1.77 (0.47–6.72)	1.58 (0.38–6.59)	**4.84** **(1.37–17.10)**
	50+ years	Ref	Ref	Ref	Ref	Ref	Ref
Gender	Male	-	-	-	**0.12** **(0.03–0.48)**	-	-
	Female	-	-	-	Ref	-	-
Personality	Extraversion	**1.34** **(1.06–1.69)**	-	1.25 (0.98–1.59)	-	-	1.20 (0.95–1.51)
	Openness	-	-	1.47 (0.98–2.22)	**2.39** **(1.48–3.86)**	1.33 (0.87–2.03)	1.50 (1.00–2.27)
	Emotional stability	-	-	-	**0.62** **(0.45–0.87)**	-	-
	Conscientiousness	-	-	-	0.68 (0.43–1.08)	-	-
	Agreeableness	-	-	-	-	-	-

Data are shown as odds ratio (95% confidence intervals). Statistically significant results are highlighted in bold text (*p* < 0.05). Variables with *p* > 0.20 were removed from the models using backward stepwise elimination. For each motivation, “not at all true” was coded as the reference category.

## Data Availability

The data presented in this study are available on request from the corresponding author. The data are not publicly available due to data governance arrangements with the IRB.

## Supplementary Materials

The following supporting information can be downloaded at https://www.mdpi.com/article/10.3390/ani13172694/s1, Supplementary Materials: Open-text responses describing additional motivations for providing foster care.
