# Supplementary material for: Understanding the Motivations of Foster Caregivers at Animal Shelters
Source: Animals (Basel). 2023 Aug 23;13(17):2694. doi: 10.3390/ani13172694 (PMC10486653; doi:10.3390/ani13172694)
Supplement: Supplementary file 1 [file animals-13-02694-s001.zip › animals-2493858-supplementary.pdf]

Open-text responses describing additional motivations for providing foster care (n=52)

A home is much better than a kennel

All of our friends foster dogs and it’s amazing so we wanted to partake to give dogs a loving home

Daughter home from college for a month and wants the companionship because she misses our dog which died about 2 years ago

Feel it’s helpful for the shelter

For my children to experience the joy of caring for animals and to model selflessness.

Fostering allows space for other animals and it is very emotionally rewarding

Fostering is an easy way I can help make the world a better place. Plus it’s so personally rewarding.

Give them an environment where they can thrive - especially if they need time to recuperate from an illness or a condition

Help a cat in need that hasn’t been adopted for a while

Help them and give them love

Helping to fill the void of losing a beloved pet

High school senior volunteer hours

I am allergic to cats, but kittens not as much- my younger son loves kittens so this is helping the kittens and him!

I don’t want an animal to suffer

I feel like I'm giving an animal the time to come into their own and make a great pet for someone.

I have a lot of love to give and want them all to find a home.

I have kids and they love animals, its a good thing to teach them to care for these little ones until they are old enough for a home. Its brought so much joy to the kids and the whole family so far. Its also just a good way to give back.

I have visited the shelter a few times and can't stand the thought of the animals stuck in kennels most of the day. They need to be outdoors and enjoying life!

I know that shelters are stressful for Animals, Stress lowers your immune system and can result in the animal getting sicker instead of better. Also, stress impacts how an animal behaves just like we are all affected by stress. In order to get a good picture of how an animal will be without the burden of stress it is important to take them out of the stressful shelter situation and provide a more normal environment where they can thrive and we can better assess what the best adoptive home for them would be for them. Lastly I want to give back all the support, love and joy that my own pets have provided to me throughout my life.

I like to work with feral kittens to improve their quality of life and their adoption potential

I love animals

I love being able to socialize and help get my foster healthy and ready for their forever homes.

I love fostering

I love kittens and I know that they are very vulnerable when they’re very young so I want to provided care for them

I love to help animals and to see them grow and blossom

I prefer to have the animals in a temporary home vs shelter

I want to give a dog a stress free environment while she or he is waiting for a home. Plus I enjoy training dogs as well

I want to help the shelters and give animals a chance

I want to provide a loving home for an animal in need

I want to provide a safe environment for fosters and puppies in need.

I wanted to care for another pet, but not take on the long term commitment.

It helps my mood become elevated and take me out of depression.

It is a good deed that is beneficial for every single party (human and animal) involved. Some animals really thrive in the space away from a shelter environment.

It is very rewarding

It's the right thing to do and I am capable of doing it.

It’s all for the animal!

Its been a wonderful lesson in compassion and empathy for my children.

Just want to help our local SPCA

Love

Love for animals

Miss having a dog in my life but don’t feel up to the commitment of permanent adoption

Model volunteer and assisting behavior for my daughter

My dog loves it too

My dog of 15 years passed away and I wasn't ready for the commitment of another dog but I missed having one.

My kids want to have a dog as a pet, but for us as a family we are not able to provide the time and care that a dog really needs

My were very Children interested

Our dog passed away and my child said he needed something to do with all his love.

Part of the solution. Prepare them for forever home, Part of the greater good.

Provide care for pets that need assistance

Seeing a feral kitten or fear aggressive dog flourish in foster care is the best feeling.

To help them thrive and find a forever home

We foster to help these neglected and abandoned pets get a better start in life to they can live healthy and happy lives.
